# Metabolically inert perfluorinated fatty acids directly activate uncoupling protein 1 in brown-fat mitochondria

**DOI:** 10.1007/s00204-015-1535-4

**Published:** 2015-06-04

**Authors:** Irina G. Shabalina, Anastasia V. Kalinovich, Barbara Cannon, Jan Nedergaard

**Affiliations:** Department of Molecular Biosciences, The Wenner-Gren Institute, The Arrhenius Laboratories F3, Stockholm University, SE-106 91 Stockholm, Sweden

**Keywords:** Uncoupling protein 1, Brown adipose tissue mitochondria, Environmental pollution, Reactive oxygen species, Membrane potential, Mitochondrial permeabilization

## Abstract

The metabolically inert perfluorinated fatty acids perfluorooctane sulfonate (PFOS) and perfluorooctanoate (PFOA) can display fatty acid-like activity in biological systems. The uncoupling protein 1 (UCP1) in brown adipose tissue is physiologically (re)activated by fatty acids, including octanoate. This leads to bioenergetically uncoupled energy dissipation (heat production, thermogenesis). We have examined here the possibility that PFOA/PFOS can directly (re)activate UCP1 in isolated mouse brown-fat mitochondria. In wild-type brown-fat mitochondria, PFOS and PFOA overcame GDP-inhibited thermogenesis, leading to increased oxygen consumption and dissipated membrane potential. The absence of this effect in brown-fat mitochondria from UCP1-ablated mice indicated that it occurred through activation of UCP1. A competitive type of inhibition by increased GDP concentrations indicated interaction with the same mechanistic site as that utilized by fatty acids. No effect was observed in heart mitochondria, i.e., in mitochondria without UCP1. The stimulatory effect of PFOA/PFOS was not secondary to non-specific mitochondrial membrane permeabilization or to ROS production. Thus, metabolic effects of perfluorinated fatty acids could include direct brown adipose tissue (UCP1) activation. The possibility that this may lead to unwarranted extra heat production and thus extra utilization of food resources, leading to decreased fitness in mammalian wildlife, is discussed, as well as possible negative effects in humans. However, a possibility to utilize PFOA-/PFOS-like substances for activating UCP1 therapeutically in obesity-prone humans may also be envisaged.

**Electronic supplementary material:**

The online version of this article (doi:10.1007/s00204-015-1535-4) contains supplementary material, which is available to authorized users.

## Introduction

Perfluorinated compounds have been utilized in a wide variety of commercial products, due to their unique surface active properties, the stability of their C–F bonds, and their thermal resistivity. These products include grease-resistant food wrapping, firefighting foams, and ski wax. Due to their stability, perfluorinated compounds accumulate in nature, and the presence of perfluorinated compounds in the environment and in bodily fluids of humans and wildlife has been well documented (Beesoon et al. 2012; Kannan et al. 2006; Theobald et al. 2011; Zhang et al. 2013). Although there is much concern that the perfluorinated compounds may present a health risk to animals and humans, there are relatively few studies concentrating on the biological effects of these compounds.

The most common perfluorinated compounds are perfluorooctanoate (PFOA) and perfluorooctane sulfonate (PFOS), i.e., the fatty acid octanoic acid structure in which all hydrogen atoms are exchanged for fluorine and the similarly altered octanoic sulfonate structure (shown in Online Resource 1). Being structurally similar to a fatty acid, the possibility may be raised that PFOA and PFOS exhibit properties akin to those of natural fatty acids—but due to their non-metabolizability, the effects would be persistent and metabolically irreversible.

A very conspicuous property of fatty acids is their ability to (re)activate UCP1, i.e., the brown fat-specific mitochondrial membrane protein that endows brown adipose tissue with its ability to produce heat, i.e., non-shivering thermogenesis (Cannon and Nedergaard 2004; Nicholls and Locke 1984). As this occurs through combustion of fat, activation of this process may result in body fat loss. Indeed, it has been shown that PFOA/PFOS supplementation to the food led to a marked decrease in mice body weight and in the mass of white adipose tissue depots (Xie et al. 2002, 2003). We have therefore examined here the possibility that PFOA/PFOS can mimic the effect of natural fatty acids by specifically activating UCP1. As this proved to be the case, the possibility exists that high environmental levels of PFOA/PFOS may affect the metabolism of animals and humans through this mechanism, resulting in decreased metabolic efficiency and potentially decreased fitness.

## Materials and methods

### Animals

UCP1(−/−) mice (= UCP1 KO mice) (progeny of those described in Enerbäck et al. (1997)) have been backcrossed to C57Bl/6 for 10 generations, and after intercrossing, they were maintained as a strain, in parallel with the wild-type C57Bl/6 mice. The mice were fed ad libitum (R70 Standard Diet, Lactamin), had free access to water, and were kept on a 12:12-h light–dark cycle, routinely at normal (22 °C) animal house temperature. The experiments were approved by the Animal Ethics Committee of the North Stockholm region.

### Mitochondrial preparation

Brown-fat and liver mitochondria were prepared as described (Cannon and Nedergaard 2008; Shabalina et al. 2010). Routinely, on each experimental day, three mice were anaesthetized for 1–2 min by a mixture of 79 % CO_2_ and 21 % O_2_ and decapitated. The interscapular, periaortic, axillary, and cervical brown adipose tissue depots were dissected out, cleaned from white adipose tissue, and pooled in ice-cold 250 mM sucrose solution. Throughout the isolation process, the tissue was kept at 0–4 °C. The tissue was minced with scissors, homogenized in 250 mM sucrose solution, filtered through gauze, and centrifuged at 8500*g* for 10 min. The supernatant with the floating fat layer was discarded. The resuspended homogenate was centrifuged at 800*g* for 10 min, and the resulting supernatant was centrifuged at 8500*g* for 10 min. The resulting mitochondrial pellet was resuspended in 100 mM KCl, 20 mM K^+^–Tes (pH 7.2), 1 mM EDTA, 0.6 % fatty acid-free BSA, and centrifuged again at 8500*g* for 10 min. The final mitochondrial pellets were resuspended by hand homogenization in a small glass homogenizer in the same medium.

Heart mitochondria were isolated principally as brown-fat mitochondria with some modifications described in Online Resource 4.

### Oxygen consumption, membrane potential, and swelling

Functions of isolated mitochondria were analyzed as described (Shabalina et al. 2004, 2013). For oxygen consumption measurements, isolated mitochondria (0.3 mg protein) were added to 1.0 ml of a continuously stirred incubation medium consisting of 100 mM KCl, 20 mM K^+^–Tes (pH 7.2), 2 mM MgCl_2_, 1 mM EDTA, 4 mM KPi, 3 mM malate, and 0.1 % fatty acid-free BSA. The substrate was 5 mM pyruvate. Oxygen consumption rates were monitored with a Clark-type oxygen electrode (Yellow Springs Instrument Co) in a sealed chamber at 37 °C. The output signal from the oxygen electrode amplifier was electronically time-differentiated and collected every 0.5 s by a PowerLab/ADInstrument (application program Chart v5.1.1.). The Chart data files were transferred to the KaleidaGraph Macintosh application and converted to absolute values, based on an oxygen content of 217 nmol of O_2_ per 1 ml of water and on the amount of mitochondrial protein used. For calculation of stable oxygen consumption rates, mean values during about 1 min were obtained from these recordings.

Mitochondrial membrane potential measurements were performed with the dye safranin O (Nedergaard 1983; Shabalina et al. 2006b) under the same conditions as those used for oxygen consumption. The changes in absorbance of safranin O were followed at 37 °C in an Olis^®^ modernized Aminco DW-2 dual-wavelength spectrophotometer at 511–533 nm with a 3-nm slit. Olis GlobalWorks™ software was used for recording and quantification.

Mitochondrial swelling was monitored as the change in absorbance at 540 nm with a 3-nm slit in an Olis^®^ modernized Aminco DW-2 spectrophotometer as described (Silva et al. 2005). The mitochondrial incubation medium and the experimental conditions were those described for oxygen consumption.

### Mitochondrial hydrogen peroxide production

Mitochondrial H_2_O_2_ net production was determined fluorometrically with the Amplex Red reagent principally as described (Shabalina et al. 2014). Mitochondria were incubated under the same conditions as those used for oxygen consumption in the presence of 50 µM palmitoyl CoA, 5 mM dl-carnitine, and 1 mM GDP. All incubations also contained 5 µM Amplex Red, 12 units ml^−1^ horseradish peroxidase, and 30 units ml^−1^ superoxide dismutase. The increase in fluorescence was detected with an EnSpire^®^ Multimode Plate Reader (PerkinElmer) in 24-well plates. The excitation wavelength was set to 563 nm, and the fluorescence emission was detected at 584 nm. The rate of H_2_O_2_ production was calculated as the change in fluorescence intensity during 2 min of the linear phase as described (Shabalina et al. 2014). Calibration curves were obtained by adding known amounts of freshly diluted H_2_O_2_ (the concentration of stock solution was checked at 240 nm using a molar extinction coefficient of 43.6) to the assay medium. The standard curve was linear in a range up till 500 nM H_2_O_2_. The calibration was performed also in the presence of GDP, substrates, PFOA, PFOS, and octanoic acid; no additions had any effect on the calibration.

### Chemicals

Fatty acid-free bovine serum albumin (BSA), fraction V, was from Roche Diagnostics GmbH. Amplex Red was from Life Technologies. Rotenone, FCCP [carbonyl cyanide *p*-(trifluoro-methoxy)-phenylhydrazone], GDP (guanosine 5′-diphosphate) (sodium salt), pyruvic acid (sodium salt), L(−) malic acid (disodium salt), palmitoyl coenzyme A (lithium salt), dl-carnitine HCl, safranin O, EDTA (ethylenediamine tetraacetic acid), horseradish peroxidase, alamethicin, PFOA (perfluorooctanoic acid), PFOS (perfluorooctane sulfonic acid) (tetraethylammonium salt), and octanoic acid (sodium salt) were all from Sigma-Aldrich Co. PFOS, PFOA, and octanoate were dissolved in 20 mM K^+^–Tes (pH 7.2) with 5 % ethanol. GDP was dissolved in 20 mM Tes (pH 7.2), and the pH of the solution was readjusted to 7.2. FCCP was dissolved in 95 % ethanol and diluted in 50 % ethanol. Ethanol in a final concentration of 0.1 % did not in itself have any effects on the parameters measured.

### Statistics

All data are expressed as means ± standard errors. Statistical analysis for the comparison of two groups was performed using Student’s *t* test.

## Results

### PFOA and PFOS activate UCP1-dependent thermogenesis in brown-fat mitochondria

**Fig. 1:**
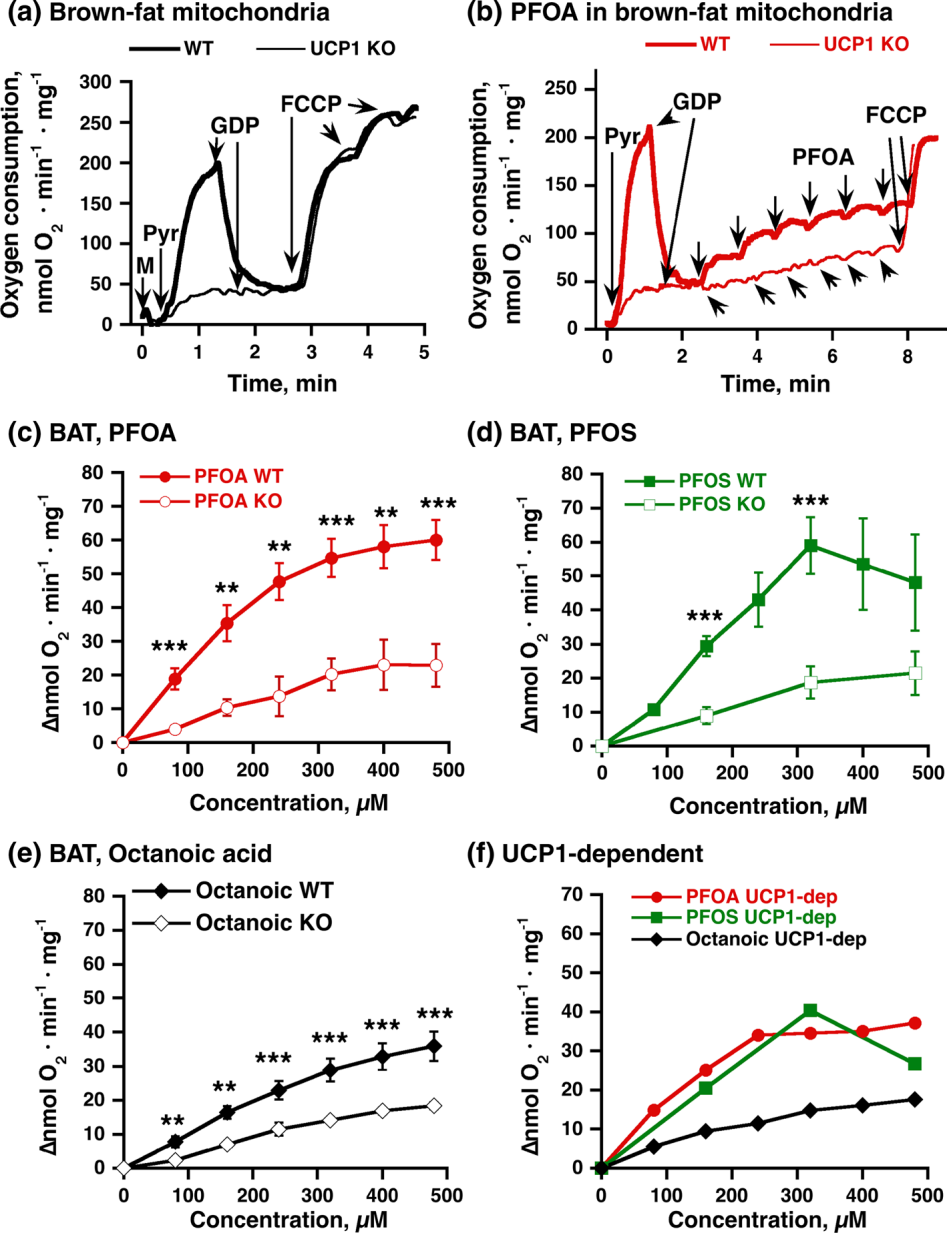
PFOA-/PFOS-stimulated oxygen consumption: dependence on UCP1. Representative traces showing the effects of FCCP (**a**) and PFOA (**b**) on oxygen consumption in brown adipose tissue mitochondria (*M*, 0.3 mg/ml) from UCP1 KO (*thin line*) or wild-type (*WT*) (*heavy line*) mice. Additions were 5 mM pyruvate (*Pyr*), 1 mM GDP, and 0.7–2.1 µM FCCP (added successively). PFOA was successively added in the concentration range 80–480 µM (each addition was 80 µM). **c** Compilation of the effect of PFOA, based on experiments as those shown in **b**. Concentration–response curve of PFOS (**d**) and octanoic acid (**e**) in brown-fat mitochondria from UCP1 KO or WT mice. Mitochondria were examined as shown for PFOA in **b**, except that not all concentrations of PFOS were examined in UCP1 KO. **f** UCP1-dependent effects of PFOA, PFOS, and octanoic acid on oxygen consumption. The values were obtained by subtraction of the means of effects in UCP1 KO mitochondria from the corresponding means of effects in wild-type mitochondria. The points in **c**–**f** are mean ± SE of 4–8 independent mitochondrial preparations for each group. *Difference between genotypes (two symbols *P* < 0.01, three symbols *P* < 0.001)

In order to examine whether the fatty acid-like perfluorinated compounds PFOA and PFOS could specifically interact with UCP1-mediated thermogenic processes in brown-fat mitochondria, we isolated brown-fat mitochondria from wild-type and UCP1 KO mice. We first confirmed the characteristic bioenergetics of these isolated mitochondria (Fig. 1a). In brown-fat mitochondria without UCP1 (*thin trace*), the basal respiration was low even in the presence of substrate in the form of pyruvate, and the addition of GDP did not affect the respiration. Subsequent addition of the artificial protonophore FCCP markedly increased the respiration. In brown-fat mitochondria with UCP1 (wild type) (*heavy trace*), the initial rate of respiration was high as expected, and it was reduced threefold to fourfold by GDP, in agreement with this high respiration being mediated by UCP1. The innately active UCP1 and the inhibition of the UCP1 activity by GDP are fundamental properties of brown-fat mitochondria, distinguishing them from mitochondria from any other tissue (Jimenez-Jimenez et al. 2006; Matthias et al. 1999; Monemdjou et al. 1999; Nicholls 1976; Shabalina et al. 2010).

The sensitivity of the mitochondria to FCCP was unaffected by the presence or absence of UCP1 (Fig. 1a). Thus, in brown-fat mitochondrial preparations such as these, wild-type and UCP1 KO mitochondria have equal oxidative capacities (reflected in the response to FCCP), and the absence of UCP1 is the limitation for thermogenesis.

Within unstimulated brown-fat cells, UCP1 is inhibited by cytosolic nucleotides (ATP, ADP, GTP and GDP) (Cannon and Nedergaard 2004; Nicholls and Locke 1984; Shabalina et al. 2004), and this inhibition is what is demonstrated with GDP in Fig. 1a. In vivo, fatty acids that are released from the stored triglycerides during adrenergic stimulation are believed to be the major reactivators of UCP1 (Cannon and Nedergaard 2004; Nicholls and Locke 1984; Shabalina et al. 2008, 2004). The following experiments were therefore designed to investigate whether PFOA/PFOS possesses the ability to overcome the GDP inhibition demonstrated above, i.e., to reactivate UCP1. We also compared PFOA/PFOS with the characteristics of octanoic acid, being the corresponding natural fatty acid, i.e., the fatty acid with the same number of carbons as PFOA/PFOS but with hydrogen replacing the fluorine molecules.

We initially observed that in brown-fat mitochondria without UCP1, PFOA induced a measurable but only minor increase in the respiration (Fig. 1b, *thin trace*). The high stimulatory effect of FCCP (Fig. 1b, *thin trace*) indicated that the mitochondria retained their high oxidative capacity (the inability of PFOA to stimulate respiration was thus not due to an inhibitory or toxic effect on mitochondrial respiration per se).

In mitochondria with UCP1 (Fig. 1b, *heavy trace*), where UCP1 activity had been inhibited by GDP, an effect of PFOA on oxygen consumption was visible already at low PFOA concentrations. The effect was much more pronounced than in the UCP1 KO mitochondria, with clear saturation kinetics. Also in these wild-type mitochondria, it was possible to expose the full oxidative capacity of the mitochondria by FCCP addition. A compilation (Fig. 1c) of experiments such as that shown in Fig. 1b clearly demonstrated that the presence of UCP1 mediated higher sensitivity to PFOA, i.e., PFOA is apparently able to reactivate UCP1.

We performed similar experiments with the analog PFOS. In the compilation in Fig. 1d, it is seen that PFOS was also able to reactivate UCP1 after GDP inhibition. However, the curve shape was not simple. It reached a maximum at 320 µM PFOS, and the effect then declined. As discussed in more detail below, this decline was related to an inhibitory effect of PFOS on the respiratory chain activity. Due to this double effect of PFOS, most of the following studies were only performed with PFOA.

To investigate whether the effect of PFOA/PFOS was mechanistically similar to that of a fatty acid, we compared the uncoupling ability of PFOA/PFOS with that of octanoic acid (Fig. 1e). As seen, also octanoic acid induced a measurable but only minor increase in respiration in brown-fat mitochondria without UCP1. Thus, there was a parallel ability (see Online Resource 2b) of octanoic acid, PFOA, and PFOS to induce such an UCP1-independent respiration. The molecular background for such fatty acid-induced respiration is not fully known. It is often referred to as a carrier-mediated uncoupling, but our earlier studies in brown-fat mitochondria indicate that it is only to the small degree mediated by activation of the adenine nucleotide translocator, in contrast to what it is in other tissues (Shabalina et al. 2006a).

Octanoic acid is a fairly potent UCP1 activator in primary brown adipocytes (Shabalina et al. 2008) and in reconstituted UCP1 systems (Winkler and Klingenberg 1994). Our results confirmed that octanoate activates UCP1 also in isolated brown-fat mitochondria (Fig. 1e).

In order to mathematically obtain the UCP1-dependent effect of PFOA, PFOS and octanoic acid, the effect of these substances in UCP1 KO mitochondria was subtracted from the effect in wild-type mitochondria (Fig. 1f). This clearly showed that the magnitude of the UCP1-dependent effects of both PFOA and PFOS was larger than the effect of octanoate (Fig. 1f). Due to their unquantified interaction with albumin, the free concentrations of the substances cannot be calculated, and true affinities can therefore not be determined; however, in nominal values, PFOA/PFOS seems to have higher affinities for UCP1 than does octanoic acid (*K*_m_ 162 for PFOA vs. 399 µM for octanoic acid). Also the maximal effect seems to be higher (*V*_max_ 51 nmol O_2_ per min per mg vs. 32) (Online Resource 2 and 3).

Thus, PFOS and PFOA specifically activate UCP1 in isolated brown-fat mitochondria, and their ability to apparently induce UCP1-mediated extra oxygen consumption (“uncoupling”) was greater than that of the fatty acid octanoate of the same chain length. Thus, exchanging the hydrogens on octanoic acid with fluorine makes the C8 carbon skeleton more competent, principally making the octanoic acid appear as a longer fatty acid—as longer fatty acids have higher uncoupling abilities (Bukowiecki et al. 1981; Fedorenko et al. 2012; Shabalina et al. 2008).

### A functionally competitive interaction between GDP and PFOA

**Fig. 2:**
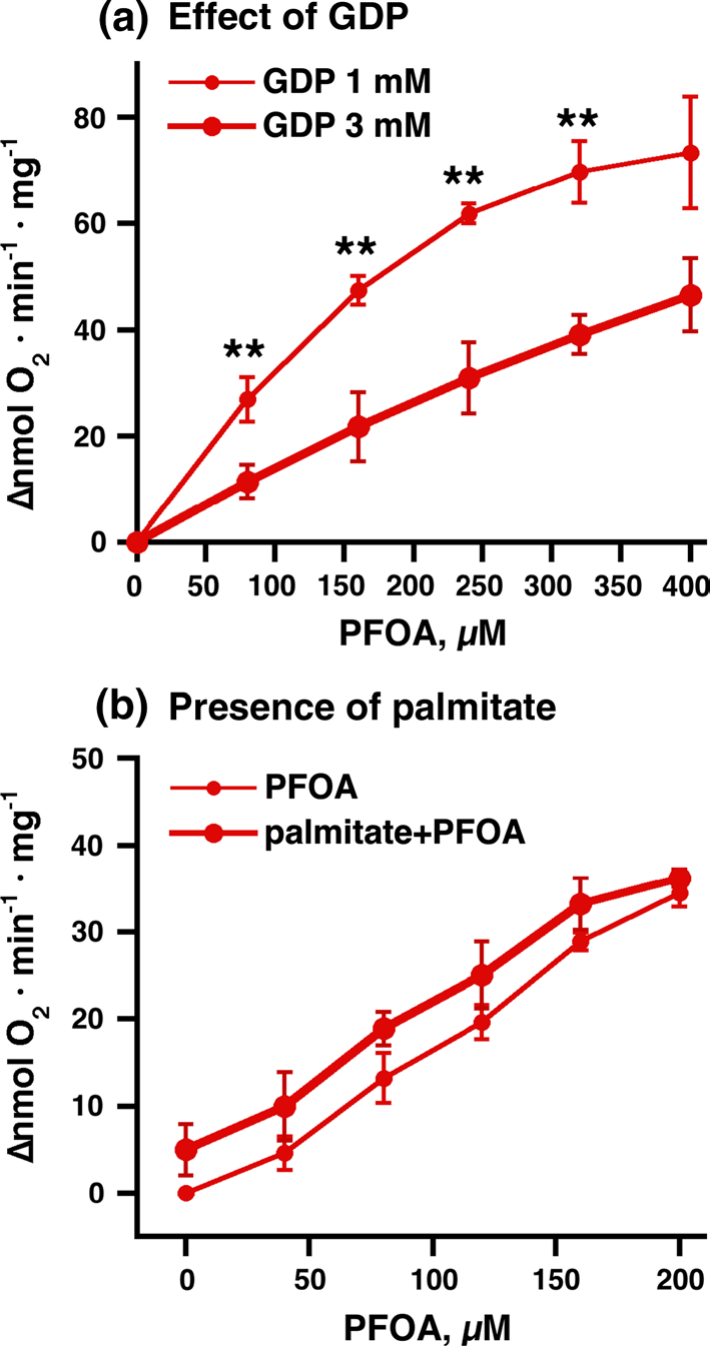
Effect of GDP and palmitate on PFOA-stimulated oxygen consumption in wild-type brown-fat mitochondria. **a** The effect of increased GDP concentration (from standard 1 mM to 3 mM) on the concentration–response curve for PFOA. Mitochondria were examined principally as shown in Fig. 1b. The points are mean ± SE of 3 independent mitochondrial preparations. ** Indicates significant difference (*P* < 0.01) with paired *t* test. **b** The effect of palmitate (20 µM) on the concentration–response curve for PFOA was also examined principally as in Fig. 1b. The points are mean ± SE of 2 independent mitochondrial preparations

The data above demonstrated that PFOA/PFOS were able to interact with brown-fat mitochondria in a UCP1-dependent manner. The data do not, however, demonstrate that PFOA/PFOS and fatty acids interact with UCP1 mechanistically at the same site. Characteristically, fatty acids reactivate UCP1 in a manner that is functional competitive with GDP (Fedorenko et al. 2012; Shabalina et al. 2004). [How this interaction occurs molecularly is not known; it would not seem to be via a direct physical competition between GDP and fatty acid on one binding site (Rial et al. 1983)]. To examine whether PFOA/PFOS shared these mechanistic interaction characteristics, we compared the reactivation competence of PFOA in the presence of 3 versus 1 mM GDP. As seen in Fig. 2a, the increased GDP level markedly influenced the kinetics. The much flatter curve shape indicated a much lower apparent affinity of PFOA for the interaction site, in agreement with a functionally competitive interaction. We therefore conclude that the interaction of PFOA/PFOS with UCP1 is of the same mechanistic nature as that of true fatty acids.

### Effect of PFOA is probably not secondary to release of fatty acids

It may be suggested that PFOA/PFOS do not interact directly with UCP1 but that the effect is secondary, i.e., through a PFOA-/PFOS-induced release of UCP1 activators, such as free fatty acids that are bound to albumin or fatty acids released from the mitochondrial membrane. Such a mechanism has earlier been proposed for the effect of acyl sulfonates on mitochondria (Jezek et al. 2006; Simonian et al. 2006). To approach this question, we titrated PFOA in the presence of added palmitate. Some of this added palmitate would bind to the albumin where it could then be competed out by PFOA. The consequence should be that the presence of palmitate should enhance the effect of a given PFOA addition.

In Fig. 2b, a comparison is shown between the dose–response curves for PFOA in the presence and absence of a small amount of added palmitate. As expected, there was a constant effect at each PFOA concentration of the addition of the small amount of palmitate, but otherwise the PFOA dose–response curve paralleled the curve in the absence of added palmitate. There was thus no enhancing effect of the presence of extra palmitate. This makes it less likely that the effect of PFOA on oxygen consumption in brown-fat mitochondria is a secondary effect; rather, PFOA/PFOS probably interact themselves directly with UCP1.

### Tissue specificity of PFOA effect

The above data indicate that PFOA interacts specifically with UCP1. As UCP1 is only found in brown adipose tissue (and in the functionally similar brite/beige cells in certain white adipose tissue depots), the implication would be that the effect should not be observable in any other tissue. To substantiate this, we tested the effect of PFOA on mitochondria isolated from heart tissue (Online Resource 4). Concerning the effect of PFOA on oxygen consumption, the trace (Online Resource 4a) showed an interaction of PFOA with these mitochondria that was very similar to that of PFOA interaction with brown-fat mitochondria lacking UCP1 (UCP1 KO), shown in Fig. 1b. Thus, no UCP1-like effects were visible in the heart mitochondria. Correspondingly, PFOA did not affect the membrane potential (see below) of these mitochondria (Online Resource 4b). Thus, there is no reason to believe that the effects of PFOA observed in wild-type brown-fat mitochondria would be paralleled by similar effects in other tissues.

### Effect of PFOA/PFOS on mitochondrial membrane potential

**Fig. 3:**
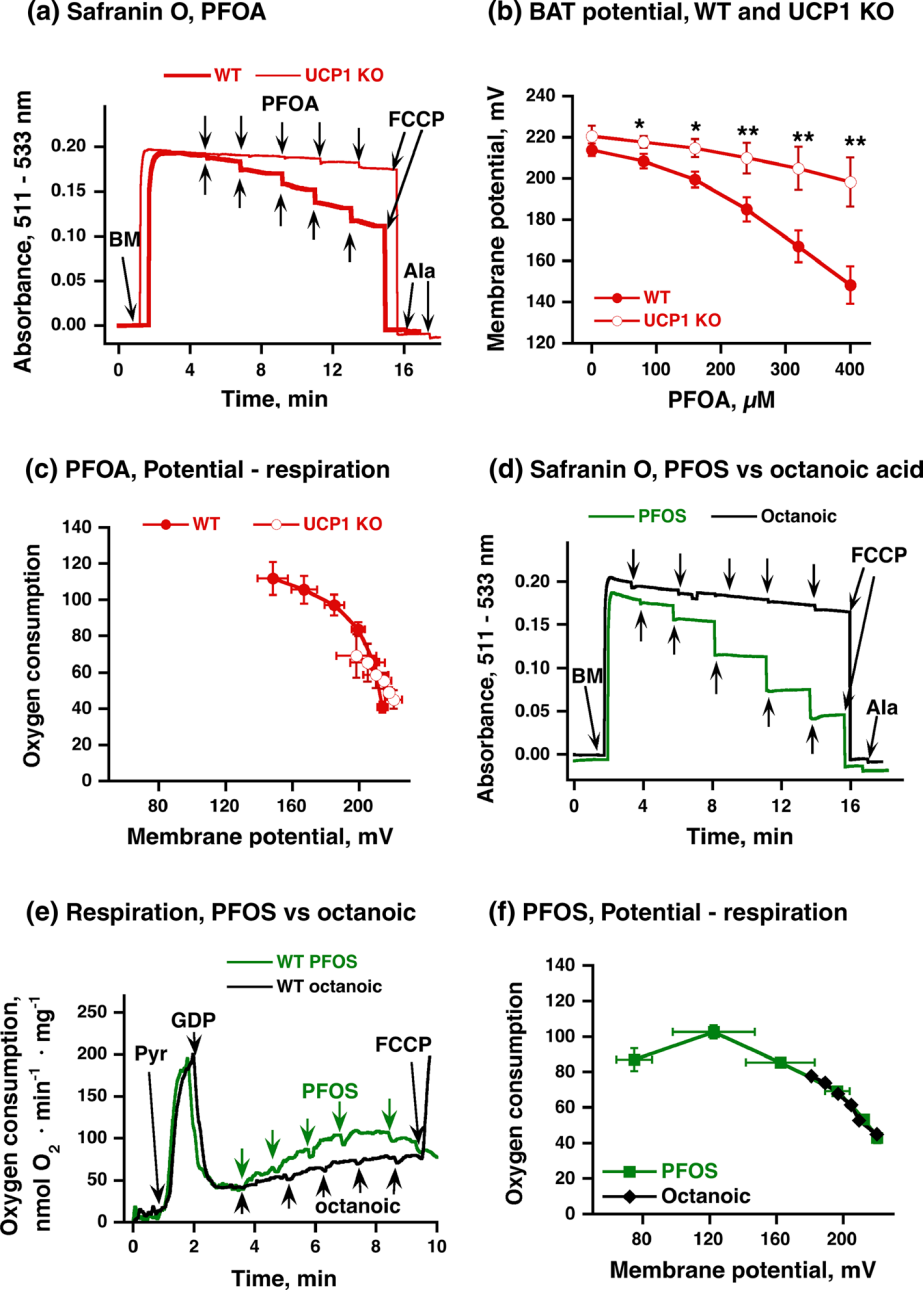
Effect of PFOA/PFOS on membrane potential in brown-fat mitochondria. **a** Representative traces showing the effects of PFOA on membrane potential (safranin O absorbance) in brown-fat mitochondria isolated from UCP1 KO (*thin line*) or wild-type (*heavy line*) mice. 5 mM pyruvate and 1 mM GDP were added before start of trace recording. Further additions in **a** were 0.25 mg/ml brown-fat mitochondria (*BM*), 2 µM FCCP and 0.02 mg/ml alamethicin (*Ala*). PFOA was added successively to obtain concentrations of 80–400 µM (each addition was 80 µM). **b** Effect of increasing PFOA concentrations on membrane potential in brown-fat mitochondria from wild-type and UCP1 KO mice. Data from experiments as in **a** were recalculated as membrane potentials (detailed in (Nedergaard 1983; Shabalina et al. 2014). **c** Oxygen consumption as an effect of membrane potential. Data from **b** were plotted with the corresponding values for oxygen consumption. To allow for direct comparisons, all corresponding results are from one experimental day, with parallel mitochondrial preparations of wild-type and UCP1 KO mitochondria, examined in parallel for respiration (as in Fig. 1) and membrane potential. The points in **b** and **c** are mean ± SE of 4 independent mitochondrial preparations for WT and 2 preparations for UCP1 KO group. In **b**, *asterisks* indicate significant differences between wildtype and UCP1 KO mitochondria (one symbol *P* < 0.05, two symbiols *P* < 0.01). **d** Representative traces showing the effect of PFOS and octanoic acid on membrane potential in wild-type brown-fat mitochondria. Conditions are as in **a**. **e** Representative traces showing the effect of PFOS and octanoic acid on oxygen consumption in wild-type brown-fat mitochondria. Conditions are as in Fig. 1b. **f** Oxygen consumption as an effect of membrane potential. As in **d**, but shown for PFOS and octanoic acid in wild-type brown-fat mitochondria. The experiment was performed twice on independent mitochondrial preparation

The increased oxygen consumption observed above in wild-type brown-fat mitochondria should, according to Mitchellian principles, be fully due to a decrease in membrane potential, as caused by increased membrane permeability for protons (equivalents). To establish whether PFOA/PFOS induced this type of classical mitochondrial uncoupling, we followed the membrane potential in these mitochondria with the safranin O technique (Nedergaard 1983), as exemplified in Fig. 3a. As seen, in the presence of GDP, the membrane potential in wild-type and in UCP1 KO brown-fat mitochondria reached the same high level (Fig. 3a), as earlier observed (Shabalina et al. 2006b, 2010). In UCP1 KO mitochondria, the signal was only weakly diminished by addition of PFOA (thin line). However, in wild-type mitochondria, the signal was markedly and dose-dependently decreased by PFOA (heavy line). FCCP and the artificial permeabilization agent alamethicin could dissipate the signal both in wild-type and UCP1 KO (Fig. 3a).

The safranin O data calculated as membrane potential are shown in Fig. 3b, c, and the relationship between membrane potential and oxygen consumption is also shown (oxygen consumption data from experiments as those in Fig. 1 but performed in parallel with the membrane potential measurements). As seen, the relationship between membrane potential and oxygen consumption was practically identical irrespective of whether PFOA exerted its small effect in UCP1 KO mitochondria or whether it functioned through activation of UCP1.

Similarly, we examined the effect of PFOS on membrane potential (Fig. 3d), in comparison with that of the natural reactivator octanoic acid. As expected, PFOS induced a much greater decrease in membrane potential than did octanoic acid. PFOS even induced a higher drop in potential than did PFOA (compare Fig. 3a and d). The reason for this became evident when we further examined the function of the mitochondria in traces such as that in Fig. 3e. It can be seen that the successive additions of PFOS initially gave rise to an increasing rate of oxygen consumption, but at higher concentrations, this was no longer the case; rather the effect became inhibitory, and FCCP can no longer overcome the inhibition. Thus, when oxygen consumption is plotted as a function of membrane potential (Fig. 3f), the relationship is initially the same for octanoic acid and for PFOS (at higher potentials). However, the curve is not monotonic, and at the highest PFOS concentrations, a further depolarization is accompanied by diminished oxygen consumption. Thus, PFOS has an inhibitory effect on the respiratory chain.

In brown-fat cells, the stimulatory effect of fatty acids to (re)activate UCP1 is strongly dependent on fatty acid chain length (Bukowiecki et al. 1981; Shabalina et al. 2008). Our observation that the magnitude of the PFOA/PFOS effect on the membrane potential was greater than the magnitude of that of the fatty acid of the same chain length (octanoic acid) was therefore intriguing. It principally could be seen to imply that the fluorinated fatty acids are experienced by UCP1 as being of a longer chain length than the native fatty acids, i.e., the uncoupling potency is not a function of the physical length of the carbon chain.

### PFOA-/PFOS-induced activation of respiration is not caused by mitochondrial disintegration

**Fig. 4:**
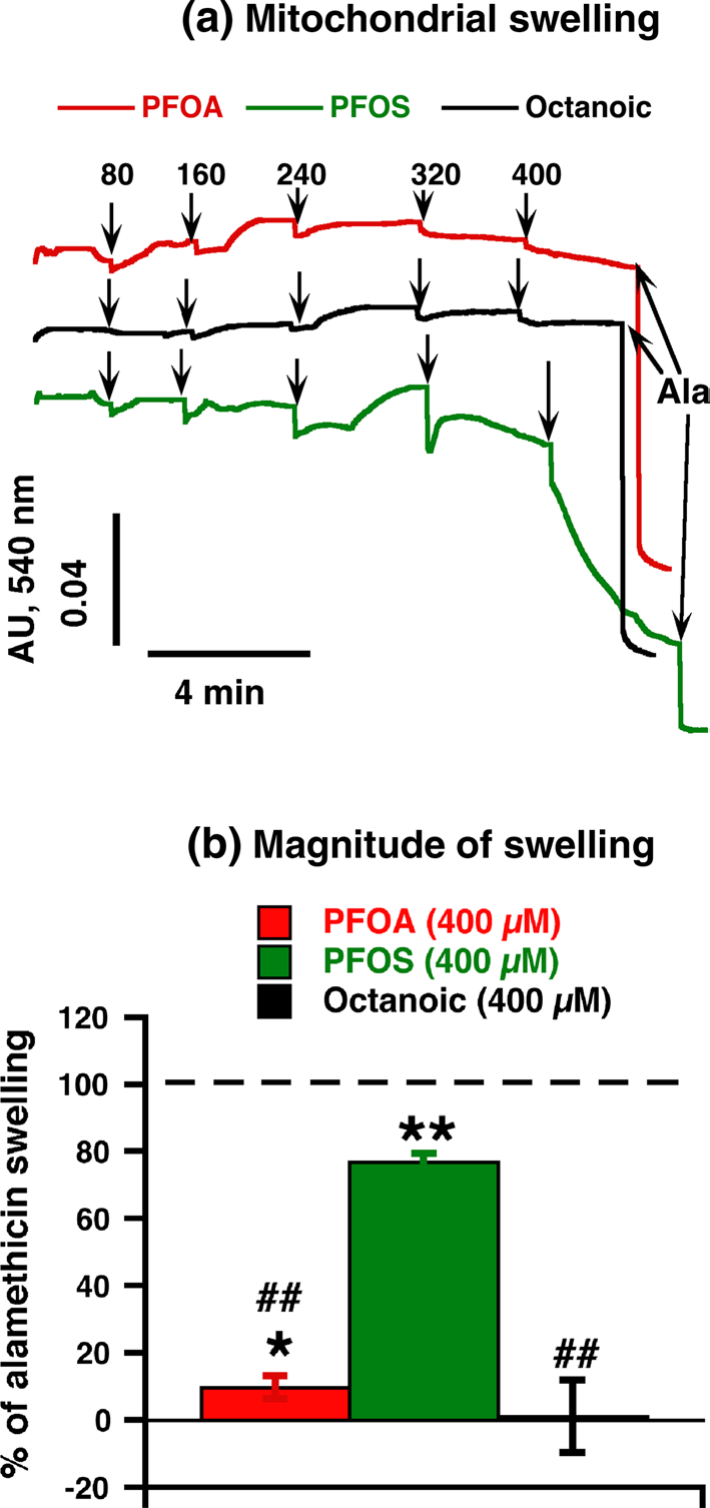
Effect of PFOA/PFOS on mitochondrial swelling in brown-fat mitochondria. **a** Representative traces showing the effects of PFOA, PFOS and octanoic acid on non-specific mitochondrial permeabilization in brown-fat mitochondria isolated from wild-type mice. Pyruvate (5 mM) and 1 mM GDP were added before the start of the trace recording. Additions were 0.25 mg/ml brown-fat mitochondria, and at the end 0.02 mg/ml alamethicin (*Ala*) (to allow for full mitochondrial permeabilization). PFOA, PFOS and octanoic acid were successively added to concentrations 80–400 µM (each addition was 80 µM and recorded during 2.0–2.5 min). To allow for direct comparisons, all traces shown are from one experimental day. **b** Quantification of the amplitude of swelling exactly 2 min after the final addition (400 µM) of PFOA or PFOS or octanoic acid. Values are indicated in percent of the maximum response [defined as the absorbance difference between the starting value (0 concentration) and the value after alamethicin addition]. The points are mean ± SE of 3–5 independent mitochondrial preparations. *Indicates difference between intact mitochondria (swelling is equal to 0) and agent; ^#^Difference between PFOS and other tested chemicals (one symbol indicates *P* < 0.05, two symbols *P* < 0.01)

An ability of PFOA/PFOS to induce mitochondrial permeabilization has been previously shown both on the cellular level (Kleszczynski et al. 2009; Panaretakis et al. 2001) and on isolated mitochondrial level (O’Brien and Wallace 2004). The UCP1-reactivating effect (“uncoupling”) observed in our experiments could thus be discussed as being due to damage of the mitochondrial membrane, associated with swelling of the mitochondria (although the tissue specificity and the UCP1-dependence of the effect demonstrated above in itself makes this less plausible). In order to clarify this, we analyzed whether PFOA/PFOS could induce swelling in wild-type brown-fat mitochondria (Fig. 4a). The addition of nominal PFOA/PFOS amounts of up to 320 µM, i.e., the concentration range necessary for the specific UCP1 activation (cf. Figs. 1, 3), had no effect on mitochondrial volume (Fig. 4a). At higher concentrations, PFOA induced only small changes in mitochondrial matrix volume (≈10 % of maximal swelling defined as that occurring with alamethicin) (Fig. 4b), whereas PFOS caused high-amplitude swelling (up to 77 %) at the highest concentration (Fig. 4b). Octanoate did not induce mitochondrial swelling (Fig. 4). Stronger detergent properties of PFOS due to the presence of sulfonate group could be responsible for stronger swelling-inducing effect of PFOS as compared to PFOA. However, clearly, the ability of PFOA/PFOS to enhance UCP1-dependent thermogenesis, which occurred in the lower concentration range, was not related to any induction of mitochondrial swelling.

In Online Resource 5, we examined whether the swelling responses were specific for the (UCP1-containing) brown-fat mitochondria or whether similar responses could be observed in liver mitochondria. We found that liver mitochondria reacted qualitatively similarly to brown-fat mitochondria (high swelling induction by PFOS, lower by PFOA, and very small effect of octanoic acid). However, liver mitochondria were much more sensitive to the agents. Our liver results showing induction of non-specific permeabilization are consistent with findings of (O’Brien and Wallace 2004; Starkov and Wallace 2002), also performed in liver mitochondria. Differences between our study and (Starkov and Wallace 2002) in PFOA concentration necessary for the effect may be due to the presence of BSA in our media for mitochondrial incubation and isolation. The much higher sensitivity of liver mitochondria than brown-fat mitochondria to the swelling-inducing agents may be related to the fact that brown-fat mitochondria contain much lower amounts of ATP synthase than does liver mitochondria (Kramarova et al. 2008). It is possible that the swelling seen is a manifestation of the activation of the membrane permeability transition pore. This pore has been suggested to be formed from dimers of the ATP synthase (Giorgio et al. 2013). Thus, lower amounts of ATP synthase in brown-fat mitochondria will confer lower sensitivity to membrane permeability transition pore-inducing agents.

### High concentration of PFOA/PFOS induces production of mitochondrial reactive oxygen species

PFOA and PFOS are competent inducers of the production of reactive oxygen species (ROS) in HepG2 cells (Panaretakis et al. 2001) associated with dissipation of mitochondrial membrane potential and apoptosis in these cells (Shabalina et al. 1999). As it has been suggested that ROS (and ROS-derived molecules) could activate UCP1 (different views on this possibility are reviewed in (Brand et al. 2004; Cannon et al. 2006), the possibility exists that the UCP1 activation could be secondary for induction of ROS production.

**Fig. 5:**
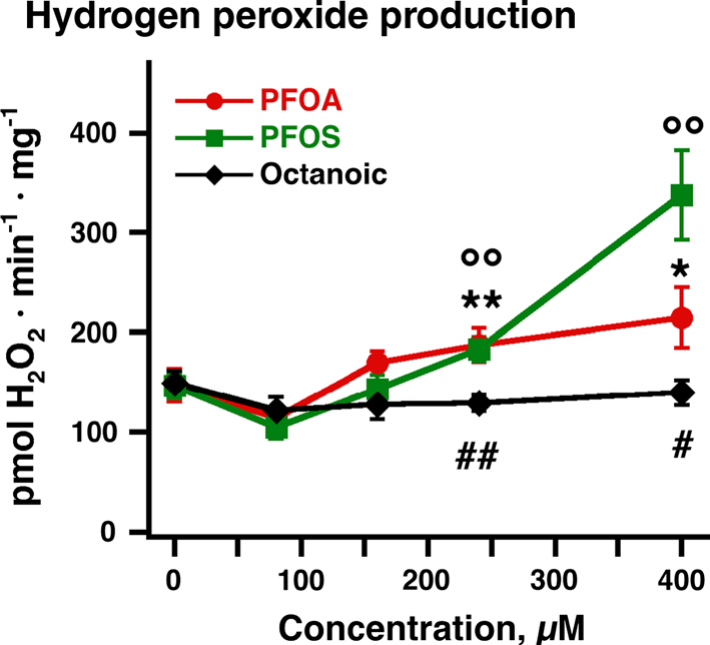
Effects of PFOA/PFOS on ROS production in brown-fat mitochondria. Mitochondrial H_2_O_2_ net production was determined fluorometrically with the Amplex Red reagent as detailed in Methods. PFOA, PFOS or octanoic acid were added in aliquots of 80 µM to wild-type brown-fat mitochondria. The points are mean ± SE of 4–7 independent mitochondrial preparations. *Difference between 0 concentration and other concentrations of PFOA; **°**Difference between 0 concentration and other concentrations of PFOS; ^#^ Indicates difference between PFOA/PFOS and octanoic acid (one symbol indicates *P* < 0.05, two symbols *P* < 0.01)

In order to examine the effect of PFOA and PFOS on ROS production in intact mitochondria, we studied brown-fat mitochondria that were actively respiring on fatty acid-derived substrate and coupled by GDP (Fig. 5). Low concentrations of either PFOA or PFOS had no effect on hydrogen peroxide production in these mitochondria. However, high concentrations of PFOA, and especially of PFOS, induced ROS production (Fig. 5). Octanoate was remarkably inert compared with PFOA and PFOS (Fig. 5). Our results indicated that PFOA and PFOS at high concentrations are indeed potent inducers of mitochondrial ROS production. It is natural to assume that the induction of mitochondrial swelling (Fig. 4), the inhibition of mitochondrial respiration (Fig. 3), and the increase in ROS production observed here are closely related phenomena: All display a similar relationship between the acids studied here, with octanoic acid being without effect, PFOA having a small effect at high concentrations, and PFOS having a large effect. It is therefore likely that the ROS production results from the inhibition of the mitochondrial respiratory chain. We have earlier demonstrated that specific inhibitor (rotenone) blockade of brown-fat mitochondrial fatty acid-supported respiration enhances/induces ROS production (Shabalina et al. 2014), and we found that a similar effect of rotenone could be observed for the PFOA-/PFOS-induced ROS production (not shown). The ROS are therefore probably derived directly from the NADH dehydrogenase complex. In view of the difference between the concentrations of PFOA/PFOS to induce thermogenesis (Fig. 1) and that needed to induce ROS production (Fig. 5), the induction by PFOA/PFOS of UCP1-dependent thermogenesis does not seem to be related to ROS production.

## Discussion

In the present investigation, we demonstrate a very specific ability of the perfluorinated fatty acids PFOA and PFOS to induce oxygen consumption in brown-fat mitochondria through activation of UCP1. The interaction with UCP1 shows mechanistic patterns, including saturation and nucleotide sensitivity, that imply that PFOA/PFOS acts through the same mechanism as do natural fatty acids in their activation of UCP1. Our observations are of relevance, both theoretically in relation to the understanding of UCP1 molecular function and practically in relation to possible PFOA/PFOS effects environmentally and for human health, negatively as well as possibly positively.

### Relation of PFOA/PFOS to molecular function of UCP1

Concerning UCP1 function, the present study is a (further) accentuation that the chemical requirements for a (re)activating agent for the protonophoric activity of UCP1 are not very high. A broad spectrum of natural fatty acids can do it (Bukowiecki et al. 1981; Shabalina et al. 2008; Tomas et al. 2004), and the present studies demonstrate that even chemically quite different fatty acid-like agents can reactivate. There is an interesting parallelism that both carbonyl fatty acids and sulfonyl fatty acids can activate UCP1, although the mechanism may be discussed (Garlid et al. 2000; Rial et al. 2004), just as can both carboxylate perfluorinated fatty acids (PFOA) and sulfonate perfluorinated fatty acids (PFOS). It is also evident that further metabolism of the (re)activating fatty acids is not necessary: neither PFOA nor PFOS can be further metabolized, just as is the case for the non-metabolizable fatty acid analog, Medica 16 (β,β’-methyl-substituted hexadecane α,ω-dicarboxylic acid) (Hermesh et al. 1998)) that is also a UCP1 (re)activator without being metabolizable (Shabalina et al. 2008). Whether the formation of the activated compound—i.e., the CoA ester—is possible for PFOA/PFOS (or Medica 16) is somewhat uncertain, and it cannot be completely excluded that the activated compounds could be formed and are those that interact with UCP1.—It is also of great interest to realize that the (re)activation potency of PFOA/PFOS is much higher than that of the corresponding non-substituted fatty acid octanoic acid. Although PFOA/PFOS have nominally higher affinities for UCP1 than octanoic acid (Figs. 1, 3 and Online Resource 2 and 3), the lower potency of octanoic acid is not due to an inability to reach saturated effects, as saturation is reached for all compounds in the present studies. Rather, the potency of a (re)activating agent is clearly more than just a function of carbon chain length.

### Possible negative environmental effects

The immediate implication of the present studies would be that PFOA/PFOS found in the environment and taken up by animals that possess brown adipose tissue could seriously affect the energy balance of these animals. This is because PFOA/PFOS would plausibly be able to activate UCP1 in situ and thus induce an unwarranted extra heat production.

Although it may spontaneously be feared that the problem with an extra, PFOA-/PFOS-induced heat production should be worst when high heat generation is needed and resources are small, this may not be the case. This is because the activity of brown adipose tissue is well regulated physiologically, and any extra heat generated, in brown adipose tissue or elsewhere in the body, will result in a compensatory decrease in the sympathetic activation of brown adipose tissue (Goldgof et al. 2014; Nedergaard and Cannon 2014; Shemano and Nickerson 1958). Instead, the problem may arise under warmer conditions where the animal would normally fully abolish cold-induced thermogenesis. If there is then an artificial extra heat production/energy utilization, the animal has no ability to compensate by decreasing physiological brown fat activation, and it will have to maintain a higher energy expenditure than that required for survival. During critical times of the year—viz. late spring when temperatures may be high but food abundance still low—this would place an extra strain on the animal which may result in diminished fitness. This may be particularly relevant for predators that experience biomagnification of perfluorinated compounds. What concentrations of PFOA/PFOS that will be encountered in nature as compared to those utilized here cannot easily be answered presently.

Such possible unwarranted activation of UCP1 would indeed also be relevant for humans. Brown adipose tissue is found and is active in newborns and infants. There is good reason to think that newborns and small infants are exposed to perfluorinated compounds. Breast milk contains perfluorinated compounds and is the main source of these in infants (Haug et al. 2011), and the concentration in breast milk (in the Stockholm area) increased during the last quarter of the last century (Sundstrom et al. 2011). There is a negative correlation between cord blood levels of perfluorinated compounds and body weight in human newborns (Apelberg et al. 2007). There is a possibility that this may be due to in utero activation of brown adipose tissue in the fetus.

### Possible therapeutic effects

Presently, for mankind in general, it is not a lack of food energy that is a major problem but rather a surplus of energy intake leading to obesity. The realization of the presence of brown adipose tissue in adult man (Nedergaard et al. 2007) has indeed promoted suggestions that a (re)activation of brown adipose tissue may be helpful in amelioration of the obesity problem (Cereijo et al. 2015; Nedergaard and Cannon 2010). We have demonstrated here that PFOA/PFOS possess the ability to directly activate UCP1 with high affinity, as compared to the effects of these compounds on mitochondrial integrity and mitochondrial ROS production. Based on the observations here on PFOA/PFOS and earlier observations on a similar effect of retinoic acid on brown-fat mitochondria (Matthias et al. 2000; Rial et al. 1999; Shabalina et al. 2008; Tomas et al. 2004), there may therefore exist a possibility to use the information on these types of compounds to enable development of substances that selectively activate UCP1, in the absence of chronic sympathetic stimulation. Such substances would not be able to recruit brown adipose tissue, and they would have to rely on available substrates for combustion. However, the amount of UCP1 could be increased through other pharmaceutical avenues. Under such conditions, such compounds would have a function, in being able to maintain the activity of the innately inactive UCP1.

## Electronic supplementary material

Below is the link to the electronic supplementary material.

Supplementary material 1 (PDF 477 kb)

Supplementary material 2 (PDF 648 kb)

Supplementary material 3 (PDF 72 kb)

Supplementary material 4 (PDF 541 kb)

Supplementary material 5 (PDF 570 kb)
