# Supplementary material for: Metabolically inert perfluorinated fatty acids directly activate uncoupling protein 1 in brown-fat mitochondria
Source: Arch Toxicol. 2015 Jun 4;90:1117–28. doi: 10.1007/s00204-015-1535-4 (PMC4830884; doi:10.1007/s00204-015-1535-4)
Supplement: Supplementary file 1 — Supplementary material 1 (PDF 477 kb) [file 204_2015_1535_MOESM1_ESM.pdf]

Metabolically inert perfluorinated fatty acids directly activate uncoupling protein 1 in brown-fat mitochondria

Archives of Toxicology

Irina G. Shabalina, Anastasia V. Kalinovich, Barbara Cannon and Jan Nedergaard
Department of Molecular Biosciences, The Wenner-Gren Institute, Stockholm University, Stockholm, Sweden. **Email: jan@metabol.su.se**

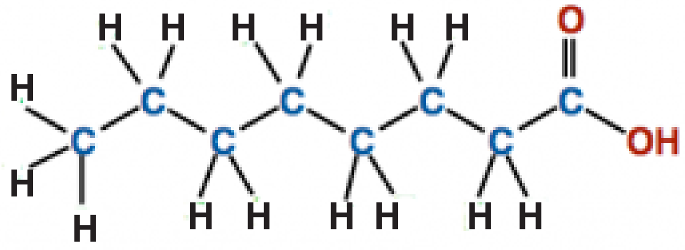

Octanoic acid

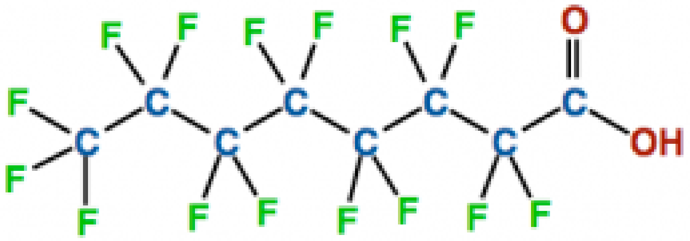

Perfluorooctanoic acid, PFOA

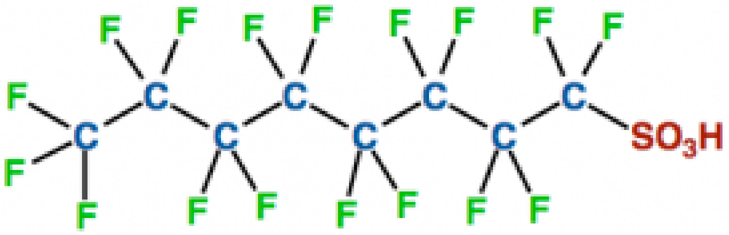

Perfluorooctanesulfonic acid, PFOS

Online Resource 1. Molecular structures of PFOA, PFOS and octanoic acid
