# Supplementary material for: Metabolically inert perfluorinated fatty acids directly activate uncoupling protein 1 in brown-fat mitochondria
Source: Arch Toxicol. 2015 Jun 4;90:1117–28. doi: 10.1007/s00204-015-1535-4 (PMC4830884; doi:10.1007/s00204-015-1535-4)
Supplement: Supplementary file 2 — Supplementary material 2 (PDF 648 kb) [file 204_2015_1535_MOESM2_ESM.pdf]

Metabolically inert perfluorinated fatty acids directly activate uncoupling protein 1 in brown-fat mitochondria
Archives of Toxicology
Irina G. Shabalina, Anastasia V. Kalinovich, Barbara Cannon and Jan Nedergaard
Department of Molecular Biosciences, The Wenner-Gren Institute, Stockholm University, Stockholm, Sweden. **Email: jan@metabol.su.se**

a. UCP1-dependent

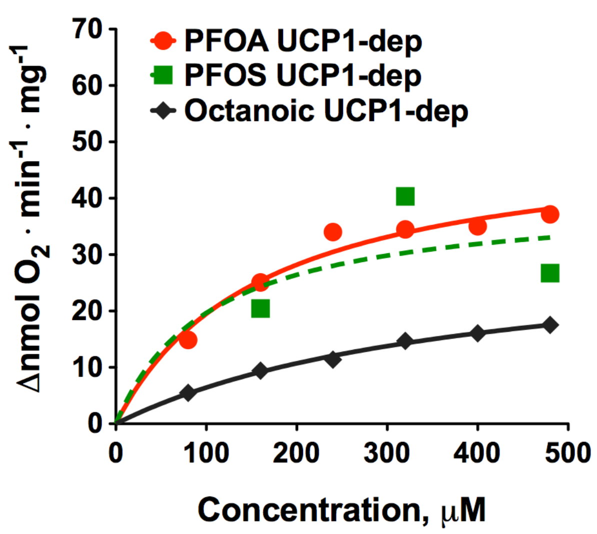

b. UCP1-independent

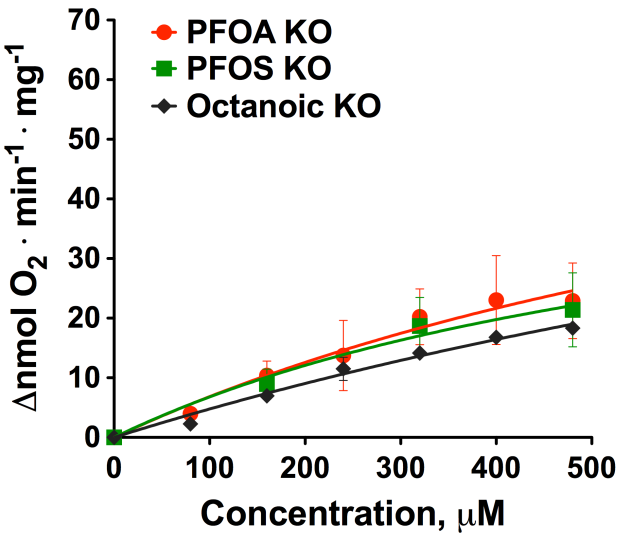

Online Resource 2

Concentration-response curve of octanoic acid, PFOA, PFOS and octanoic acid in brown-fat mitochondria

(**a**) UCP1-dependent concentration-response curves presented on Fig. 1f were analyzed with the best fit option of the GraphPad Prism application for adherence to simple Michaelis-Menten kinetics.
(**b**) UCP1-independent concentration-response curves presented on Fig. 1c, d, and e were analyzed with the best fit option of the GraphPad Prism application for adherence to simple Michaelis-Menten kinetics.
