# Supplementary material for: Metabolically inert perfluorinated fatty acids directly activate uncoupling protein 1 in brown-fat mitochondria
Source: Arch Toxicol. 2015 Jun 4;90:1117–28. doi: 10.1007/s00204-015-1535-4 (PMC4830884; doi:10.1007/s00204-015-1535-4)
Supplement: Supplementary file 3 — Supplementary material 3 (PDF 72 kb) [file 204_2015_1535_MOESM3_ESM.pdf]

Metabolically inert perfluorinated fatty acids directly activate uncoupling protein 1 in brown-fat mitochondria

Archives of Toxicology

Irina G. Shabalina, Anastasia V. Kalinovich, Barbara Cannon and Jan Nedergaard

Department of Molecular Biosciences, The Wenner-Gren Institute, Stockholm University, Stockholm, Sweden. **Email: jan@metabol.su.se**

Online Resource 3

Parameters of Michaelis-Menten kinetics fitting of concentration-response curves of PFOA, PFOS and octanoic acid in brown-fat mitochondria

Parameters	UCP1-dependence	PFOA	PFOS	Octanoic acid
K_m µM	UCP1-dependent (WT – UCP1 KO)	162	N/C	399
	UCP1-independent (UCP1 KO)	676 ± 110	243 ± 162	2503 ± 12#
V_{max} $\frac{nmol\ O_2}{min \cdot mg}$	UCP1-dependent (WT – UCP1 KO)	51	N/C	32
	UCP1-independent (UCP1 KO)	49 ± 10	24 ± 6	92 ± 7#

UCP1-independent K_m and V_{max} are mean ± S.E. obtained from analysis of each individual concentration-response curve for PFOA, PFOS, and octanoic acid for the best fit option of the GraphPad Prism application for adherence to simple Michaelis-Menten kinetics; n = 2-3.
UCP1-dependent K_m and V_{max} are necessarily based on the subtracted mean values only but are thus indirectly based on 5-6 independent preparations.
N/C: not calculable (concentration-response curve for PFOS does not fit to simple Michaelis-Menten kinetics)
# indicates statistically significant difference between octanoic acid and PFOA ($P < 0.05$)
