# Supplementary material for: Metabolically inert perfluorinated fatty acids directly activate uncoupling protein 1 in brown-fat mitochondria
Source: Arch Toxicol. 2015 Jun 4;90:1117–28. doi: 10.1007/s00204-015-1535-4 (PMC4830884; doi:10.1007/s00204-015-1535-4)
Supplement: Supplementary file 4 — Supplementary material 4 (PDF 541 kb) [file 204_2015_1535_MOESM4_ESM.pdf]

Metabolically inert perfluorinated fatty acids directly activate uncoupling protein 1 in brown-fat mitochondria
Archives of Toxicology
Irina G. Shabalina, Anastasia V. Kalinovich, Barbara Cannon and Jan Nedergaard
Department of Molecular Biosciences, The Wenner-Gren Institute, Stockholm University, Stockholm, Sweden. **Email: jan@metabol.su.se**

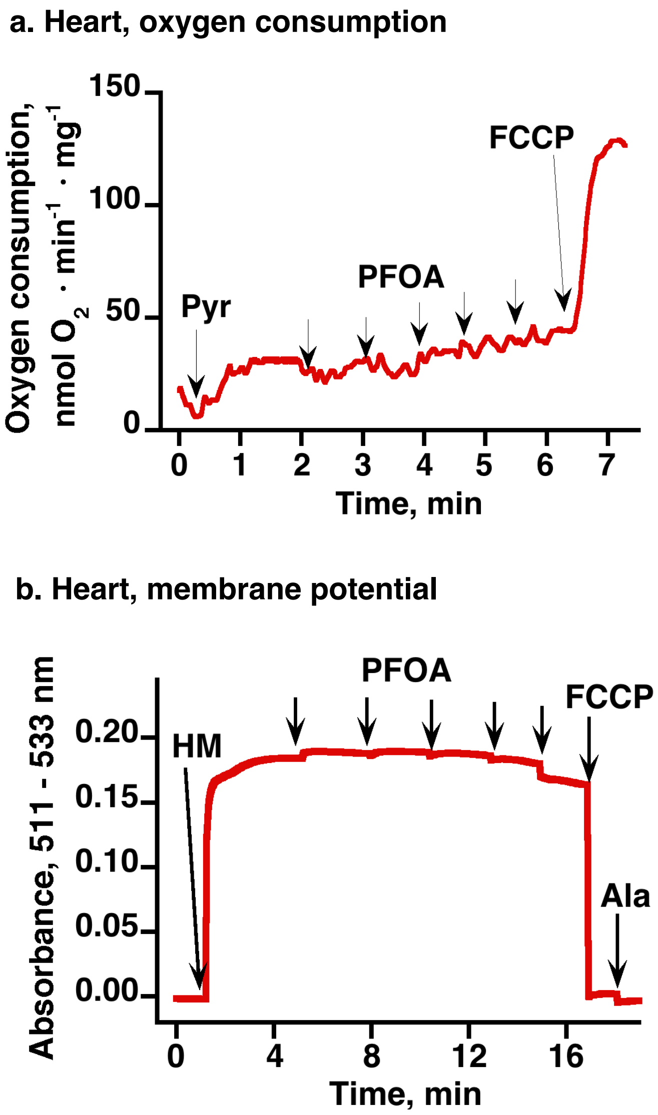

Online Resource 4

Effects of PFOA on oxygen consumption and membrane potential in heart mitochondria.

Representative traces showing the effect of PFOA on oxygen consumption (a) and membrane potential (b) in heart mitochondria isolated from wildtype mice. Additions were 0.25 mg/ml heart mitochondria (HM), 5 mM pyruvate (Pyr), 1.2 µM FCCP and 0.02 mg/ml alamethicin (Ala). PFOA was successively added in the concentration range 80–400 µM (each addition was 80 µM). To allow for direct comparisons, traces shown are from one experimental day, examined in parallel for respiration and membrane potential. The experiment was performed twice on independent mitochondrial preparations, with similar outcomes. Heart mitochondria were isolated by differential centrifugation principally as described for brown fat mitochondria (see Materials and Methods) except that the isolation medium was consisting of 100 mM sucrose, 50 mM KCl, 20 mM K-TES, 1 mM EDTA and 0.2 % (w/v) fatty-acid-free BSA and the heart homogenate was exposed to nagarse (1 mg per g tissue) treatment during 2-3 min before centrifugation. Incubation medium was consisting of 100 mM sucrose, 20 mM K-Tes (pH 7.2), 50 mM KCl, 2 mM $MgCl_2$, 1 mM EDTA, 4 mM KPi, 0.1 % fatty-acid-free BSA. and 3 mM malate.
