# Supplementary material for: Metabolically inert perfluorinated fatty acids directly activate uncoupling protein 1 in brown-fat mitochondria
Source: Arch Toxicol. 2015 Jun 4;90:1117–28. doi: 10.1007/s00204-015-1535-4 (PMC4830884; doi:10.1007/s00204-015-1535-4)
Supplement: Supplementary file 5 — Supplementary material 5 (PDF 570 kb) [file 204_2015_1535_MOESM5_ESM.pdf]

Metabolically inert perfluorinated fatty acids directly activate uncoupling protein 1 in brown-fat mitochondria
Archives of Toxicology
Irina G. Shabalina, Anastasia V. Kalinovich, Barbara Cannon and Jan Nedergaard
Department of Molecular Biosciences, The Wenner-Gren Institute, Stockholm University, Stockholm, Sweden. **Email: jan@metabol.su.se**

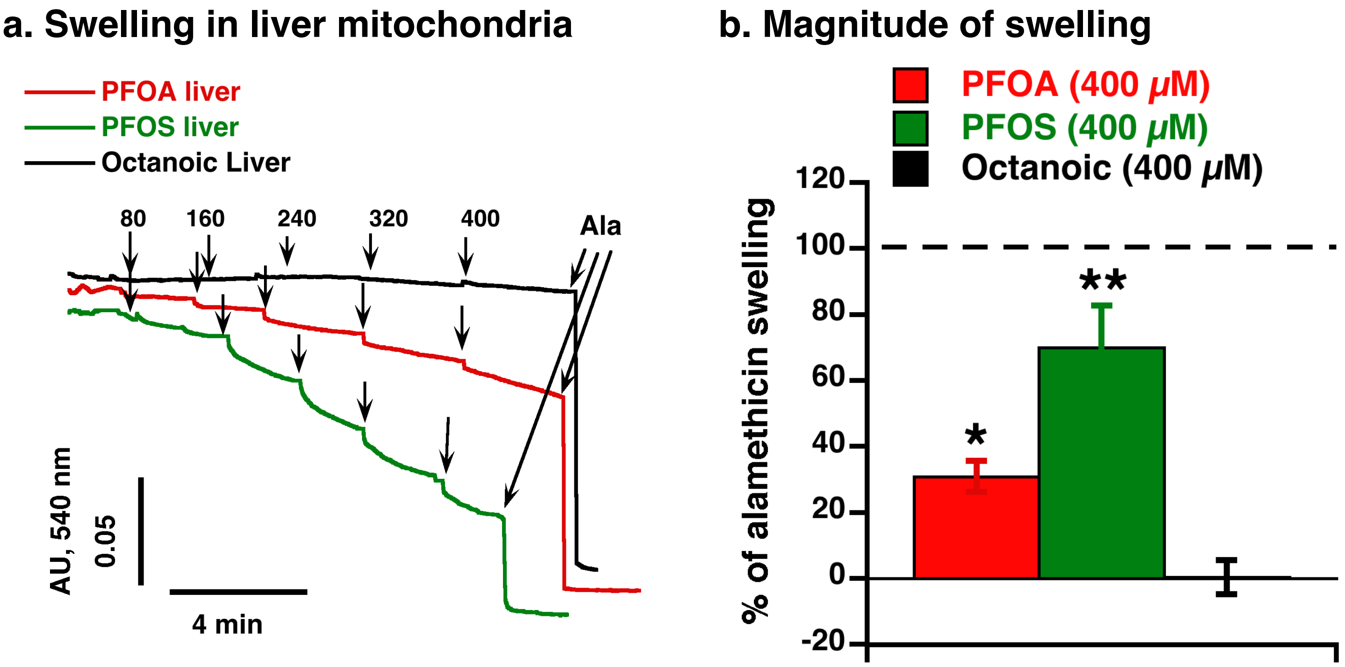

Online Resource 5

Effect of PFOA/PFOS on mitochondrial swelling in liver mitochondria.

(a) Representative traces showing the effects of PFOA, PFOS and octanoic acid on nonspecific mitochondrial permeabilisation in liver mitochondria isolated from wildtype mice. Glutamate (5 mM) and 2 mM malate were added before the start of the trace recording. Additions were 0.25 mg/ml liver mitochondria, and at the end 0.02 mg/ml alamethicin (Ala) (to allow for full mitochondrial permeabilisation). PFOA, PFOS and octanoic acid were successively added to final concentrations 80–400 μM (each addition was 80 μM and recorded during 2.0-2.5 min).

(b). Quantification of the amplitude of swelling 2 min after the final addition (400 μM) of PFOA or PFOS or octanoic acid. Values are indicated in percent of the maximum response (defined as the absorbance difference between the starting value (0 concentration) and the value after alamethicin addition). The points are means ± S.E. of 3-5 independent mitochondrial preparations. Difference between octanoic acid and other tested chemicals (* indicates $P < 0.05$, ** - $P < 0.01$)
